# Performance Comparison of Flow-Through Optofluidic Biosensor Designs

**DOI:** 10.3390/bios11070226

**Published:** 2021-07-07

**Authors:** Joel G. Wright, Md Nafiz Amin, Holger Schmidt, Aaron R. Hawkins

**Affiliations:** 1 Electrical and Computer Engineering, Brigham Young University, 450 Engineering Building, Provo, UT 84602, USA; joel.g.wright@byu.edu; 2 Electrical and Computer Engineering, University of California, 1156 High Street, Santa Cruz, CA 95064, USA; mdamin@ucsc.edu (M.N.A.); hschmidt@soe.ucsc.edu (H.S.)

**Keywords:** optofluidic, hydrodynamic focusing, lab-on-a-chip, biosensor, liquid-core waveguide, fluorescence

## Abstract

Optofluidic flow-through biosensors are being developed for single particle detection, particularly as a tool for pathogen diagnosis. The sensitivity of the biosensor chip depends on design parameters, illumination format (side vs. top), and flow configuration (parabolic, two- and three-dimensional hydrodynamic focused (2DHF and 3DHF)). We study the signal differences between various combinations of these design aspects. Our model is validated against a sample of physical devices. We find that side-illumination with 3DHF produces the strongest and consistent signal, but parabolic flow devices process a sample volume more quickly. Practical matters of optical alignment are also discussed, which may affect design choice.

## 1. Introduction

Optofluidic biosensors utilizing fluorescing molecules to tag pathogens in a lab-on-a-chip platform are in development for clinical use [1]. These biosensors operate by passing biosample targets tagged with fluorescing markers through an intersecting exciting light. The markers fluoresce in this light, and the fluorescence can be detected and measured by a photodetector as a signal for target presence. Targets detectable by this biosensor platform include antigens, cancer biomarkers, liposomes, nucleic acids, proteins, ribosomes, and virions [1,2,3,4,5,6].

Notably, this biosensor platform has been used to detect the H1N1 virus [7], various antibiotic-resistant bacterial genes [5], and the recent SARS-CoV-2 virus [1]. Meena et al. demonstrated the detection of *E. coli*, *E. aerogenes*, *K. pneumonia*, KPC, and VIM with accuracies of 91.6%, 90.2%, 85%, 90%, and 90%, respectively; their tests yielded an average signal-to-noise ratio (SNR) of 39. Furthermore, they demonstrated the simultaneous detection of multiple targets [5]. Stambaugh et al. demonstrated biosensing tests in which influenza A antigens were detected at a rate of 5.7 events per second, and SARS-CoV-2 antigens were detected at a rate of 2.6 events per second; these events were observed with an SNR of 80 [1]. The goal of continued biosensor development is to increase the sensitivity of the device, which for this platform means designing the sensor to collect and transmit a fluorescent signal both high in intensity and with low variance.

Some developments in the field of biosensing include the use of nanopores to isolate bio-targets for specific examination [8,9,10,11]. The nanopore allows for the electrical detection of targets, including single-molecule targets, and offers some benefit of controlling the delivery of the sample to the sensing structure of the device [8,11]. Nanopore applications can even trap complex nucleic molecules and facilitate translocation of their subcomponents [9]. Additionally, coupling electrical sensing from the nanopore structure with optical sensing can allow for multiple signals from the same target to be detected and even label-free optical detection [10,11]. However, in optical detection systems, it is useful to have the capability to predict how a sensitive a device can be given multiple simultaneous optical and fluidic considerations, and the intent of this work is to explore this capability through modeling and simulation.

The optofluidic biosensors described here consist of several components: a channel through which the fluid biosample is conducted, a waveguide and/or a facet for guiding excitation light to the channel, and a waveguide for collecting the signal fluorescence and guiding it to the off-chip photodetector. Because of these several integrated components, designing the biosensor requires a comprehension of optics, waveguiding, fluorescence, and fluid dynamics. Previous work has been done in the development of a model which integrates each of these concepts, and that work showed that variations in design parameters result in differing biosensor performances [12]. This model can be used to emulate existing device designs and predict the performance of designs yet to be fabricated. However, the model was only previously used to describe optofluidic biosensors with a side excitation format, planar with the sensor chip. This was examined for parabolic fluid flow where a biosample could occupy nearly any position in the channel cross section. To be more complete, other excitation and fluid flow conditions must be incorporated into the model.

**Figure 1:**
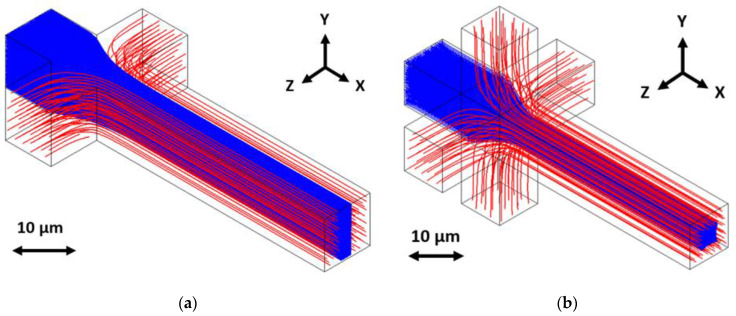
Conceptual illustration of hydrodynamic focusing methods, with the sample streams given in blue and the buffer streams given in red: (**a**) A two-dimensional hydrodynamic focusing (2DHF) method has two inlets coming from the side to focus the sample horizontally; (**b**) A three-dimensional hydrodynamic focusing (3DHF) method has two inlets coming from top, bottom, and sides to focus the sample horizontally and vertically. This figure is cited from Hamilton et al. in *Micromachines* [15] under Creative Commons Attribution License.

**Figure 2:**
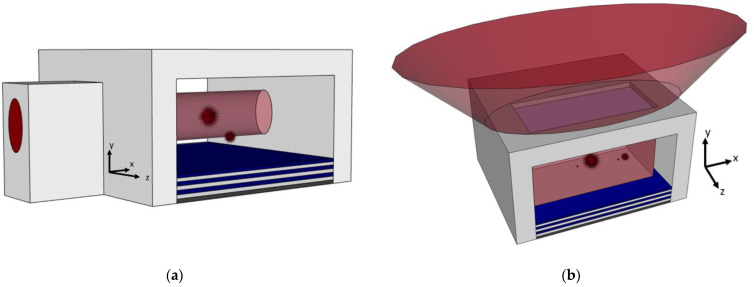
Conceptual examples of the two illumination formats under study, with the example illuminating light given in red: (**a**) A side-illuminated device has an optical mode guided by a ridge waveguide to the ARROW channel; (**b**) A top-illuminated device has a window through an opaque covering over the ARROW channel, and an illuminating light is spread over the window.

Two other developments in the optofluidic biosensor field show promise for greater sensitivity and increased functionality. Hydrodynamic focusing is one such development, in which the flowing biosample is constrained, either two- or three-dimensionally, to a stream in a region smaller than the channel cross-section, illustrated in Figure 1 [13,14,15]. The intention for confining flow in this way is to produce fluorescence signals with less variation and force biosample velocity to be more uniform. Another development being included in biosensor designs is a top-down illumination scheme, an alternative to planar side illumination [16,17]. In this setup, the top of the biosensor is made opaque, excepting windows above the channel, and a source excitation light is directed onto these windows from above. This bypasses the need to fine-align an optical fiber to an excitation waveguide, giving greater alignment tolerance and quicker practical diagnosis. A comparative illustration is given in Figure 2.

This article will show how the expected fluorescence signal for a collection of biosamples will compare for biosensors that incorporate different flow focusing and illumination modalities. First, we will outline the basic fabrication process of the standard optofluidic biosensor and explain how the process differs if hydrodynamic focusing is added. Next, we will show the model to be validated by previous physical experiment. Then the model will make performance predictions for an array of biosensor designs. Standard parabolic, two dimensional hydrodynamic focusing (2DHF), and three dimensional hydrodynamic focusing (3DHF) flow regimes will each be examined with standard side illumination and newer top illumination excitation. After these model simulations are calculated, we will discuss the various design performances and offer commentary on which would offer the best clinical function.

## 2. Optofluidic Biosensor Fabrication

The biosensor is built around liquid-filled anti-resonant reflecting optical waveguides (ARROWs) which also act as the channels through which the sample flows. This is a waveguide with a liquid core that still guides light with the core having a lower refractive index than bounding channel walls. The ARROW relies on a periodic series of dielectric layers grown on top of a silicon substrate [18,19]. These layers consist of three pairs of SiO_2_ and Ta_2_O_5_, with SiO_2_ being deposited on the substrate first. These ARROW layers allow for guiding within the channel.

**Figure 3:**
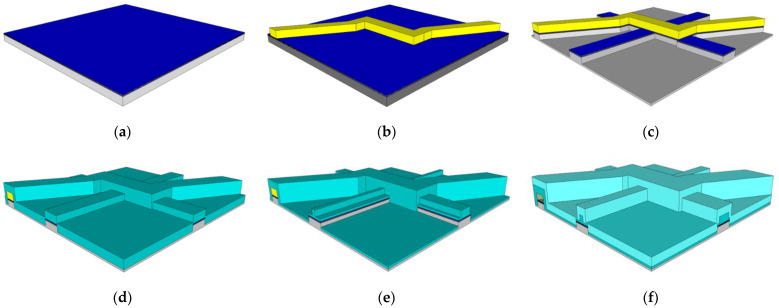
(**a**) ARROW layers are grown on top of a silicon substrate. (**b**) SU8 photoresist is coated on the ARROW layers, is patterned and developed by photolithography to leave the sacrificial core for the ARROW channel. (**c**) A pedestal for the core and pending solid waveguides is etched into the silicon bulk. (**d**) A layer of 1.51 refractive index silicon dioxide is grown over the whole device. (**e**) Ridges are etched into the oxide layer to make the excitation and collection waveguides. (**f**) A second silicon dioxide layer, 1.46 refractive index, is grown over the whole device, and the sacrificial core is etched out to leave the ARROW channel clear for fluid flow.

The fabrication process begins with a silicon wafer on which the ARROW layers are grown (Figure 3a). The wafer is then coated with a film of SU8-10 photoresist. This is a negative photoresist, and by way of photolithography it can be patterned and developed to form a rectangular structure (Figure 3b). This developed SU8 forms a sacrificial core around which the channel is built. This core is typically 12 µm wide and 5–6 µm high, depending on intended design parameters.

Next, a pedestal is patterned by photolithography. This pattern is made with a positive photoresist, AZ4620, and developed to expose the top of the SU8 core and the pedestal area. Nickel is evaporated onto the wafer, and the remaining photoresist is lifted off with acetone; this leaves a nickel mask on the core and desired pedestal area. The pedestal is then etched in a deep reactive ion etching (DRIE) process, etching through the ARROW layers and into the silicon bulk, 6 µm from the surface of the ARROW layers. The nickel mask is wet etched away, leaving the device to appear as illustrated in Figure 3c.

At this point, a layer of SiO_2_, 1.51 refractive index and 6 µm thick, is grown over the whole device by plasma enhanced chemical vapor deposition (PECVD), shown in Figure 3d. This layer forms the channel walls and the material from which the solid ridge waveguides are formed. Then, the ridge waveguides are etched by the same process as the pedestal, illustrated in Figure 3e. A cladding layer of SiO_2_, 1.46 index and 6 µm, is grown over the whole device to protect the inner waveguides from atmospheric water absorption. Finally, the SU8 core is etched out with a solution of sulfuric acid and hydrogen peroxide. The final biosensor device appears as shown in Figure 3f.

**Figure 4:**
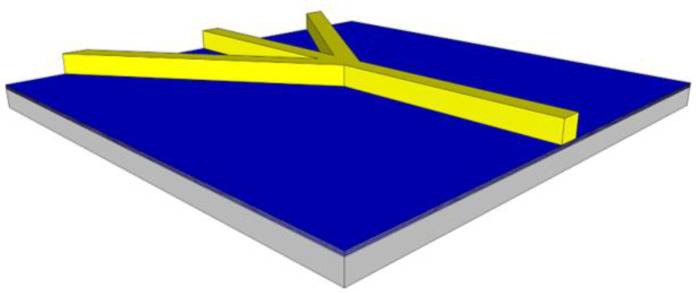
The modified 2DHF device requires a modified core step to allow two inlets on the side of the main sample channel. Subsequent fabrication steps require corresponding modifications to the photolithography patterns.

**Figure 5:**
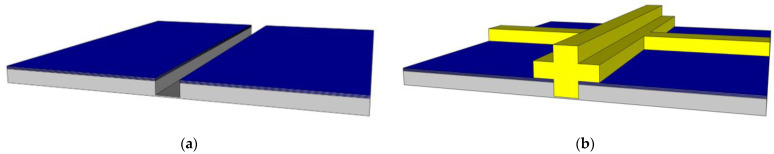
Modified steps for a 3DHF device: (**a**) A trench is etched into the substrate bulk; (**b**) SU8 photoresist is layered and developed in a two-step process. The first SU8 layer fills the trench and covers the substrate. This layer will be patterned and developed to leave the channel core and most of the buffer inlet cores. The second step places another layer on the device, which is patterned and developed to leave a raised rectangular structure over the trench.

Variations for the 2DHF and 3DHF biosensor designs require a more sophisticated core than the standard design. The 2DHF core requires simply a change in photolithography patterns (from the SU8 core to the ridge mask) to allow for side channels containing a buffer fluid to intersect with the primary sample channel, focusing the sample fluid horizontally, shown in Figure 4. This is a simple example of focusing through sheath flow. The 3DHF design requires etching into the substrate prior to the placement of the SU8 core [20]. A trench is etched into the ARROW layers and substrate by DRIE. The wafer is then coated with SU8 and patterned and developed to make the standard channel and the inlet buffer channels. A second SU8 coating is then applied, patterned, and developed to leave a higher and more narrow rectangular structure on top of the inlet SU8 core, shown in Figure 5. This allows for the inlet buffer fluid to compress the intersecting sample fluid from the sides, top, and bottom. The compressed sample stream then occupies a smaller area within the channel cross section. Again, this focusing technique relies on sheath flow, both horizontally and vertically.

**Figure 6:**
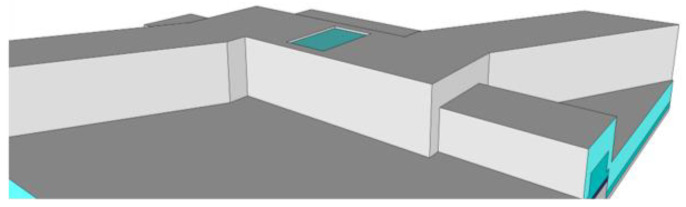
A close-up view of the window for top-illumination. A layer of aluminum is deposited over the whole device, and a window is milled through the aluminum, leaving the transparent oxide exposed.

In order to build biosensors with top illumination, rather than side illumination requires additional steps after the sacrificial SU8 core has been etched out. A layer of optically opaque material, such as aluminum, is deposited over the biosensor device [12]. Windows through the aluminum and over the ARROW channel are either patterned using lithography and etching or directly milled by focused ion beam, leaving the transparent silicon dioxide exposed, as shown in Figure 6.

## 3. Model Theory Overview

The optofluidic biosensor as described above integrates several concepts. The core of the model is the volume where the excitation light and the ARROW channel intersect, which is referred to as the excitation region (ER). A brief overview of the model shall be given here, but a more extensive description has been previously published [12].

The first component of the model for consideration is the excitation light source. In side-illuminated devices, an optical mode is guided to the channel by a solid ridge excitation waveguide. This mode has an intensity profile that can be approximated with a Gaussian distribution. The model’s mode profile does not fill the entire cross section of the intersected liquid core, but the illuminated volume will have a high optical intensity. The mode wavelength should be such that the mode will excite the fluorescent marker selected. For most fluorescence sensing, this will mean a visible wavelength like 633 nm and fluorescent dyes like Cy5 and Alexa Fluor 647 (ThermoFisher Scientific^®^, Waltham, MA, USA).

Top-illuminated devices are illuminated by a laser beam focused by a lens onto the device [17]. It is assumed that the power intensity is approximately uniform in a single window; this assumption will be explained in the next section. This illumination format will illuminate the entire channel cross section.

As the bioparticle crosses the ER, the optical power incident on it can be calculated as cumulative across the width of the optical profile. This accumulation is a function of how the particle intersects the optical intensity profile and the amount of time crossing the ER. To calculate the amount of time a particle spends crossing the ER, a fully integrated laminar flow profile is used to calculate the flow velocity profile of the channel cross section [21,22]. Absent hydrodynamic focusing, this results in a parabolic flow profile, with the peak velocity in the center of the channel. Using this flow velocity profile and the depth of the ER (the width of the optical profile), we calculated the amount of time a particle is illuminated and fluorescing. This time is a function of the particle’s position in the channel cross section and is greatest close to the inner channel surfaces.

The time profile is multiplied by the cumulative power to produce an excitation energy profile. This energy profile corresponds with the amount of fluorescence a particle emits based on its position in the channel cross section. To calculate the amount of fluorescence that will transmit down the length of the ARROW and be collected by the collection ridge waveguide, a sweep of finite differential time domain (FDTD) simulations was done according to particle position in the channel cross section. This collection efficiency profile indicates the ARROW is the most transmissive when the particle fluoresces in the center of the channel profile. This collection efficiency profile is multiplied with the excitation energy profile to make the ER profile. This profile relates the amount of fluorescence energy that will be collected according to the bioparticle’s channel cross section position [12]. The side illumination format has a mode with an intensity peak that intersects the collection efficiency peak, whereas the top illumination format illuminates less collection-efficient regions of the ARROW channel.

When simulating the performance of a biosensor design, the ER profile is sampled randomly for position according to a number of particles in the simulated sample. The amount of fluorescence energy for each sampling is then calculated into a number of photons per unit time (counts/0.1 ms) to give the signal value used in biosensor testing. A distribution of signals is then produced for the number of sampled particles. The mean of this signal distribution and its coefficient of variance (CV), defined as the quotient of the standard deviation and the mean, define the biosensor design’s sensitivity and consistency [12].

## 4. Validation of Side and Top Illuminated Formats

To validate the model with two possible illumination formats, the model’s calculations were compared to physical tests of both side and top illuminated devices [17]. To perform this validation, we compared the mean signal of each device to the optical power incident on the ARROW channel. These power-normalized signals were then compared between illumination formats.

**Figure 7:**
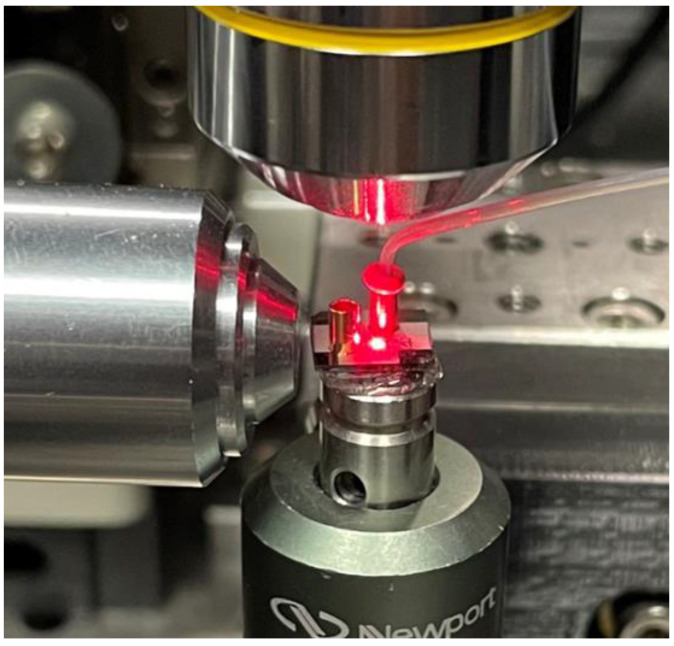
Test setup of an optofluidic biosensor chip in a top-illumination format. One copper bead is used as a sample reservoir and is attached to the channel inlet. A second copper bead connects the channel outlet to a vacuum to instigate fluid flow. A laser illuminates the channel excitation region from above (a side-illuminated chip will have a fiber connected to the laser and connects it to a planar ridge waveguide). On the left is an objective that collects the fluorescence signal and focuses it onto the APD.

In the physical tests done for validation, the biosensor chip was integrated into a setup that includes an exciting laser, fluid flow actuator, and fluorescence detector. An example of the test setup geared for top-illumination is shown in Figure 7. Copper beads were attached to the two open ends of the ARROW channel; one is used as a sample reservoir, and the other was attached to a vacuum that actuates the fluid flow. The vacuum used in this setup is a VPES3 1/4HP vacuum pump (VIOT^®^, Champaign, IL, USA) which creates a negative pressure on the channel outlet.

A 633-nm laser is used to illuminate the excitation region. This laser can be guided to the side facet of a solid-ridge waveguide or focused onto the channel in a top-illumination format. A SPCM-AQR-14-FC avalanche photodiode (Perkin Elmer^®^, Waltham, MA, USA) was used to detect the fluorescence signal [23]. The avalanche photodiode (APD) registers the signal by putting a 2.5 V reverse bias across the sensing photodiode. This bias is sufficiently strong that an incident photon will generate a charge carrier in the diode junction which in turn will generate secondary charge carriers, increasing the current. This amplified current allows for a single photon to be detected. The current’s magnitude is then measured to correspond to the number of incident photon counts. This particular APD has a 65% detection efficiency for photons in the 650–700 nm wavelength spectrum and a low-noise dark photon count rate of 0.01 counts/0.1 ms.

The side illuminated biosensor excited passing particles with a seven-spot multi-mode interferometer (MMI) waveguide, used for signal multiplexing [24]. For testing, fluorescent beads (ThermoFisher Scientific^®^ FluoSpheres™ 625/645 Crimson) 0.2 µm in diameter were used as targets as a stand-in for bioparticles. These beads will provide a sufficient substitute for bioparticles in sensitivity testing as both would be subject the same ER factors of optical excitation, flow rate, and fluorescence collection efficiency. The laser power was set to 5.3 mW, with 151 µW on each spot for a total channel-incident power of 1.057 mW. The mean flow velocity was 0.935 cm/s. There were 136 beads detected, and the mean signal from this device was 161 counts/0.1 ms. Normalized for power, the mean signal for the side illuminated device was 152 counts/(mW·0.1 ms).

The top illuminated device had seven 14 × 4 µm^2^ windows, also creating a seven-spot excitation pattern in the channel. The excitation laser was transmitted through an objective lens system that focused it into an elliptical spot. The spot had diameters of 85 µm long and 22 µm wide. Because the 12 µm channel width fits well within the short diameter of 22 µm, the optical intensity was assumed to vary negligibly across the channel width. The laser power was set to 19 mW; an average 553 µW was incident on each window for a total of 3.871 mW. The mean flow velocity was 1.50 cm/s. There were 136 bead particles detected, and the mean signal was 195 counts/0.1 ms. The normalized mean signal was 50 counts/(mW·0.1 ms). The side-illuminated power-normalized signal was 3× greater than the top-illuminated signal.

**Figure 8:**
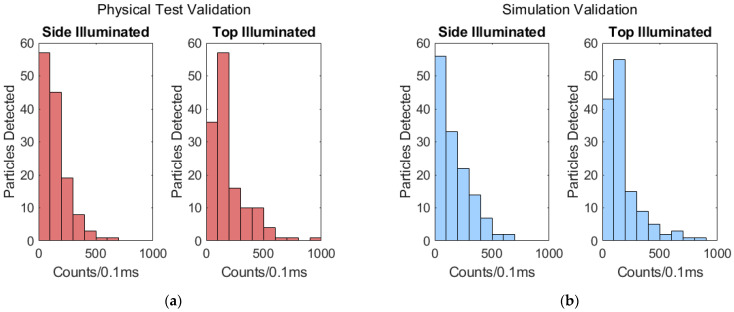
Comparison of signal distributions for model validation: (**a**) The physical test signal distributions for both side- and top-illuminated devices; (**b**) The simulated signal distributions for side- and top-illuminated models.

Validation was done by adjusting the power settings of each side- and top-illuminated models to match the mean signals of the physical tests. In these cases, waveguide and facet transmissions are also assumed uniform, and it is simply the input optical powers that are adjusted. To accurately simulate the physical tests, we replicated the multiplexing, seven-spot excitation regions in the model. To result in a mean signal of 161 counts/0.1 ms that matches the physical test results, the side-illuminated model’s power was set to 26.4 µW; the power-normalized mean signal was therefore 6098 counts/(mW·0.1 ms). Likewise, the top-illuminated model’s power was set to 80 µW to result in a mean signal that matches the physical test result of 195 counts/0.1 ms; this signal was power-normalized to give 2438 counts/(mW·0.1 ms). The ratio of these two power-normalized signals is 2.50; this ratio is lower than the respective ratio in the physical tests, but still comparable. The signal distributions of the validation tests and simulations are given in Figure 8, showing a similarity in distribution after the mean signals were matched.

There is a large difference in the input power used in the experiment versus the model in order to produce the same mean output fluorescence power (counts/0.1 ms). This difference comes because the model only accounts for light being coupled into the liquid core waveguide. Unaccounted for are losses at the interface between the liquid core and a solid core waveguide, losses along the length of these waveguides, and loss at the chip facet. Because of these unknown loss factors, we are more interested in comparing the output power distributions for a collection of particles. The plots in Figure 8 have purposely been scaled to represent the same number of particles. As can be seen, whilst the experiment results do not match the model exactly, they are very close; the intersection between the side-illuminated distributions is 85%, and the top-illuminated intersection is calculated to be 92% [25]. They are close enough to give us confidence regarding using the model to make further projections for other device designs. Our earlier publication regarding the model an array of side-illuminated devices provides additional confidence in this validation’s accuracy [12].

## 5. Comparing Designs by Illumination Format and Flow Regime

### 5.1. Preliminary Information

To make a comparison between each modelled device studied here, most parameters had to be kept constant across all design variations. Parameters were chosen based on commonly used fabrication dimensions and test constraints. The total optical power incident on the channel was constant across all variations. For conceptual simplicity in the simulations, a single excitation region was simulated in each case. The ARROW channels were given constant height and width dimensions, and the flow velocity profile was kept constant across all variations. In each study case, 1000 particles are simulated at random locations in the channel flow cross section (for the hydrodynamic focusing cases this meant shrinking the cross section that sample particles could occupy). The signals simulated in the following device projections were based on fluorescence generated and collected in the ARROW’s excitation region. The design parameters relevant to this study are listed in Table 1.

### 5.2. Flow Regime Cross Sections

Differences in the models include the excitation format and the excitation region cross section of each flow regime example. To keep the premises of the modeled projections straightforward, the samples were illuminated by a single mode spot or a single window for the respective designs [17]. The dimensions of the sampleable ER profiles were chosen based on previous work in parabolic, 2DHF, and 3DHF flow regimes [7,20]. In the case of the 2DHF designs, the focusing in the model was modified to keep the focused sample in the horizontal center of the channel and to have a focused sample stream width of 4 µm. The 3DHF designs constricted the ER both horizontally and vertically. The constricted sample stream cross section had a width of 3 µm and a height of 4 µm. Table 2 lists ER cross sections by flow regime and the time it would take to pass 1000 particles through the respective ERs if contained in an initial sample volume of 100 nL.

### 5.3. Side-Illuminated Designs

**Figure 9:**
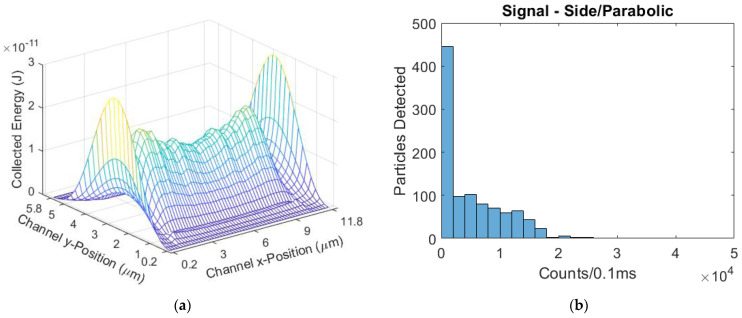
(**a**) The modeled excitation region for the side-illuminated biosensor with a parabolic flow regime; (**b**) The simulated signal distribution.

The excitation region profile of the side-illuminated, parabolic flow regime device has the greatest variation in sampleable energy values. The Gaussian-like mode intensity has its peak in the center of the channel height, intersecting the ARROW collection efficiency peak, seen in Figure 9a. This results in a high number of low energy values to be sampled, and a relatively small number of medium and high energies sampled. This distribution is shown in Figure 9b. The mean signal for this design was 5040 counts/0.1 ms, with a CV of 1.071.

**Figure 10:**
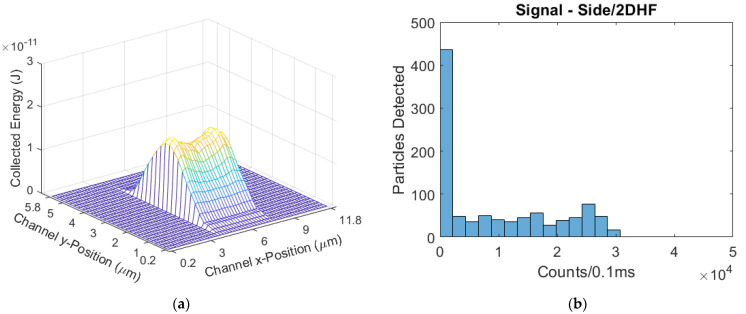
(**a**) The modeled excitation region for the side-illuminated biosensor with a 2DHF flow regime; (**b**) The simulated signal distribution, with a skew further to the right than the parabolic flow device.

The side-illuminated, 2DHF device had an ER that has similar variance of energy values to the parabolic regime, as the restricted sampleable cross section was focused horizontally and not vertically (Figure 10). The mean signal was 9499 counts/0.1 ms, an 88% increase over the parabolic-flow design. The CV was 1.042, a 3% decrease from the parabolic-flow design, which is not a significant improvement by itself.

**Figure 11:**
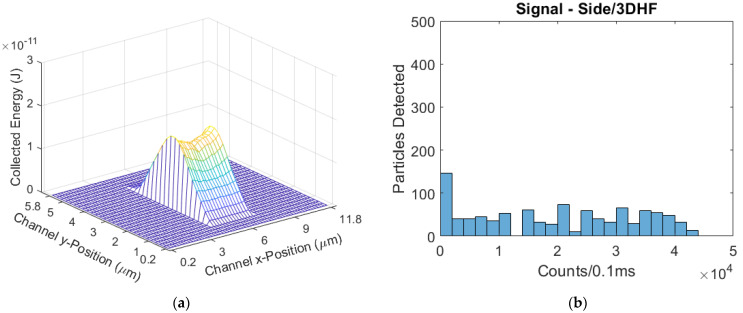
(**a**) The modeled excitation region for the side-illuminated biosensor with a 3DHF flow regime; (**b**) The simulated signal distribution, with the furthest-right skew of all devices in the side-illumination format.

The side-illuminated, 3DHF device visibly has the least variance in its ER profile as the simulated sample streams have been focused horizontally and vertically, shown in Figure 11. The mean signal was 19,901 counts/0.1 ms, with a CV of 0.672. The focusing of the sample streams into both the optical intensity and collection efficiency peaks raised the mean signal 295% and reduced the CV by 37% from the parabolic-flow design. Table 3 lists the signal statistics at the end of this section.

### 5.4. Top-Illuminated Designs

Unlike a side-illuminated device, top-illuminated devices have a uniform optical intensity distribution in the ER. Variance in the ER’s energy profile is due to variance of time in the excitation region. As the sample streams become hydrodynamically focused, the time variance decreases, decreasing ER energy variance.

**Figure 12:**
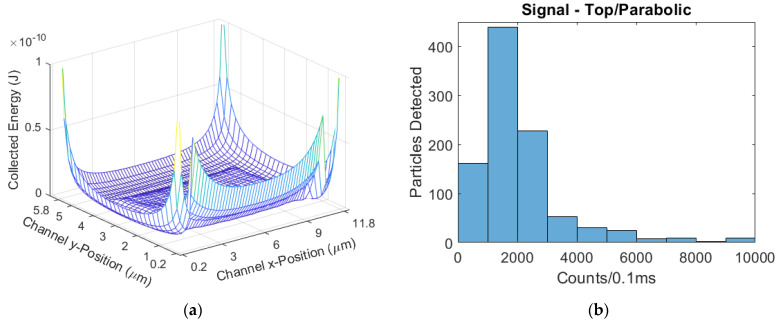
(**a**) The modeled excitation region for the top-illuminated biosensor with a parabolic flow regime; (**b**) The simulated signal distribution.

**Figure 13:**
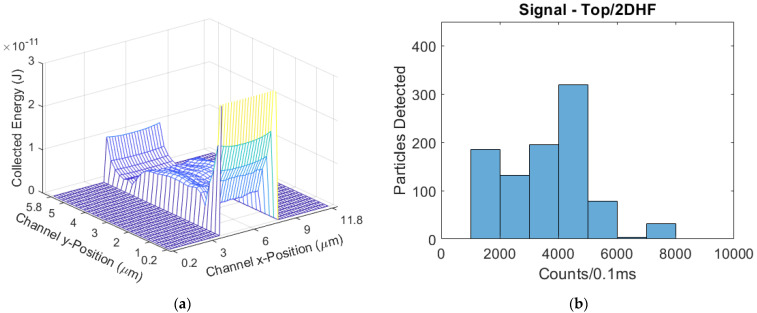
(**a**) The modeled excitation region for the top-illuminated biosensor with a 2DHF flow regime; (**b**) The simulated signal distribution, with a skew further to the right than the parabolic flow device.

**Figure 14:**
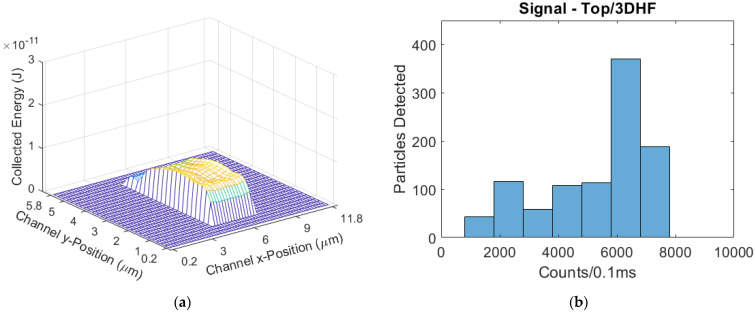
(**a**) The modeled excitation region for the top-illuminated biosensor with a 3DHF flow regime; (**b**) The simulated signal distribution, with the furthest-right skew of all devices in the side-illumination format.

The parabolic-flow design (Figure 12) had a mean signal of 2575 counts/0.1 ms. The parabolic CV was 1.190. The 2DHF design (Figure 13) had a mean signal of 4243 counts/0.1 ms, a 65% increase from parabolic; the 2DHF CV was 0.748, a 37% decrease. The 3DHF design (Figure 14) had a mean signal of 5300 counts/0.1 ms, a 106% increase over the parabolic flow regime; the CV was 0.332, a 72% decrease from parabolic.

### 5.5. Optimization of Side-Illuminated, 3DHF Design

Further commentary on the array of designs is provided later, but at this point we can see the side-illuminated/3DHF design has the highest mean signal and second smallest CV. While this designed ER was based in part on a hydrodynamically focused cross-section from a previous work, the opportunity presents itself to optimize this design for better signal strength and variance. The hydrodynamically focused cross-section in the ER was varied according to degree of vertical focus and horizonal position within the channel.

**Figure 15:**
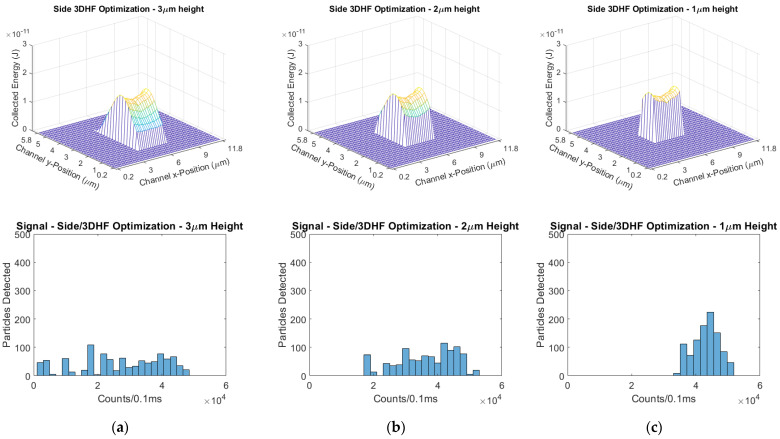
Excitation region profiles and signal distributions of a side/3DHF design with decreasingly focused cross-section height. Compare to the 4 µm height shown in Figure 10a. (**a**) 3 µm height, (**b**) 2 µm height, and (**c**) 1 µm height.

Keeping the horizonal position of the focused cross-section centered in the channel, we shortened the cross-section height in 1 µm intervals. The 4 µm height was previously simulated in Section 5.3, and the 3 µm, 2 µm, and 1 µm heights were simulated subsequently. As the simulated hydrodynamically focused cross section gets shorter, the mean signal increases and the signal CV decreases to 9%. However, it is estimated that a sample of 1000 particles will require 797 s to pass through this excitation region. The simulated ERs and signal distributions are shown in Figure 15, and the signal means and CVs are compiled in Table 4.

**Figure 16:**
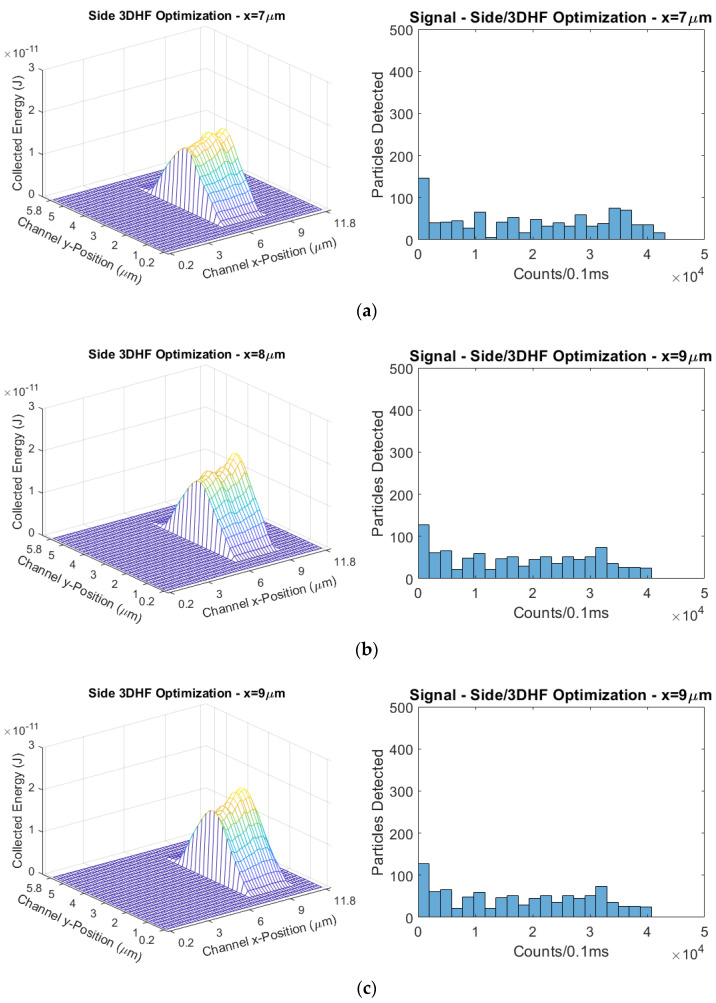

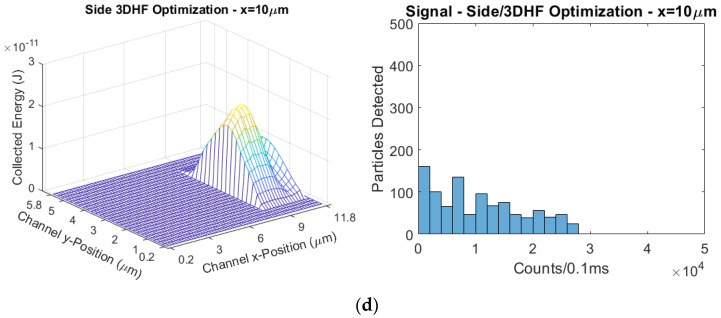
Excitation regions and signal distributions of simulated hydrodynamically-focused cross-sections shifted horizontally from the channel center (x = 6 µm). Compare to the cross-section centered at x = 6 µm shown in Figure 10a. (**a**) x = 7 µm; (**b**) x = 8 µm; (**c**) x = 9 µm; (**d**) x = 10 µm.

The other parameter sweep done to optimize the side-illuminated/3DHF design was the x-position of the hydrodynamically focused cross-section within the ARROW channel. The cross-section dimensions were kept constant in this case, and the center of the cross-section was simulated at 1 µm intervals from x = 6 µm (horizontal center) to x = 10 µm. The cross-section width being 3 µm, the sweep brings the side of the cross-section to be half of a micron from the ARROW sidewall. In this parameter sweep, the successive simulated iterations show a decrease in mean signal and an increase in signal CV. Therefore, trying to shift the focused stream laterally from the center offers no benefit to the sensor design. The simulated excitation regions and their corresponding signal distributions are given in Figure 16, and the mean signals and CVs are compiled in Table 5.

## 6. Discussion of Simulation Results

In each illumination format, the 3DHF devices had the highest mean signals and least variance relative to signal. The side-illumination format also was shown to have higher mean signals compared to the top-illumination format, but the top-illuminated devices had lower CVs in hydrodynamically-focused flow regimes. The best simulated device in terms of signal strength and lowest CV was the side-illuminated 3DHF design.

While 3DHF devices had the best results in both illumination formats, they also required the longest time to detect 1000 particles, 3× over the parabolic flow devices pre-optimization and 11× when optimized for signal. This should be considered for clinical application. If time is short in a clinical setting, utilizing a parabolic flow device could be judged a suitable compromise in time saved and sufficient accuracy. The 3DHF design also has the most steps involved in its fabrication process.

While the side-illuminated devices had the best signal performance, the practical matter of aligning and butt-coupling a fiber laser source to the excitation ridge waveguide will add time to the test procedure. The advantage of the top-illumination format would be the greater alignment tolerance of a window or series of windows under a laser beam spread by an objective lens. While the signal would be weaker, a top-illuminated 3DHF device would feature the signal with the least variance relative to signal strength given the previously demonstrated hydrodynamic focusing capability [20]. The optimized side-illuminated/3DHF design would have the lowest CV of the simulated designs if a 1µm-high focused sample stream is attained.

It should also be noted that these simulations were done with a 0.2 µm-diameter bead as the analyte, which would be the fluorescence emissions equivalent of multiple fluorophores. During a test procedure in which pathogens are detected, the targets would be marked with one of a variety of fluorophore types. Common fluorophore dyes for sensing are Cy3 and Cy5. These are families of molecules made from cyanine chemical chains [26]. There are many other commercial dyes used, but their chemical structures are proprietary. Another small fluorescing analyte is the quantum dot, which is a semiconductor crystalline particle (tens of nanometers in scale) which can be designed for specific absorption/emission wavelengths [27].

Nucleotide targets are commonly tagged with either an intercalating dye or a molecular beacon. Intercalating dyes are used when the test requires a high density of fluorophores on the target to emit a larger amplitude of fluorescence, but this method is not target-specific when other molecules are present [28]. Molecular beacons are quenched fluorophores attached to a fabricated oligonucleotide sequence; this sequence will bond with its complementary target, and the fluorophore becomes unquenched [29]. This method is target-specific, but will have a lower fluorescence amplitude than the intercalating dye method. Additionally, dyes are reported to be attachable to single proteins, bypassing the need bond with target nucleotide sequences [1]. The number of fluorophore molecules of these biosensing exercises will result in a reduced signal magnitude compared with the larger fluorescent beads.

## 7. Conclusions

In this study, we outlined the uses and operation of the optofluidic biosensor device as well as how they are fabricated. Using physical tests of devices, we validated a mathematical model and projected how an array of design variations would perform in signal magnitude and variance. Using the simulated projections, we found that the side-illuminated 3DHF design has the best signal performance, but at a cost of slightly increased fabrication complexity and longer clinical test times. The parabolic flow-regime devices will pass the biosample through the ER in a shorter test time than the hydrodynamically focusing devices. These operational differences do allow clinicians options for testing based on the scenario presented to them.

## Figures and Tables

**Table 1:** General model parameters constant across all studied design iterations.

Parameter	Value
Input Power	1 mW
Channel Height	6 µm
Channel Width	12 µm
Mean Flow Velocity	2 cm/s
Bead Diameter	0.2 µm
Excitation Wavelength	633 nm
Emission Wavelength	645 nm

**Table 2:** Excitation region cross sectional area and test time for each design variation.

	Flow Regime		
	Parabolic	2DHF	3DHF
Excitation Region Cross Section (µm^2^)	65.0	22.4	12.0
Estimated Sample Test Time (s)	73	168	237

**Table 3:** Summary of simulation signals for each design model.

		Flow Regime		
		Parabolic	2DHF	3DHF
Side Illumination	Mean Signal (counts/0.1 ms)	5040	9499	19,901
	CV	1.071	1.042	0.672
Top Illumination	Mean Signal (counts/0.1 ms)	2575	4243	5300
	CV	1.190	0.748	0.332

**Table 4:** Results of hydrodynamically-focused cross-section height optimization.

	Cross-Section Height			
	4 µm	3 µm	2 µm	1 µm
Mean Signal (counts/0.1 ms)	19,901	27,121	36,488	42,932
CV	0.672	0.481	0.249	0.091

**Table 5:** Results of hydrodynamically-focused cross-section horizontal position optimization.

	Cross-Section Horizonal Position				
	6 µm	7 µm	8 µm	9 µm	10 µm
Mean Signal (counts/0.1 ms)	19,901	19,643	20,295	17,730	10,653
CV	0.672	0.680	0.684	0.681	0.720
